# It Isn’t Your Town—It’s You

**Published:** 1916-03

**Authors:** 


### EDITORIAL DEPARTMENT

MRS. F. E. DANIEL, Publisher and Managing Editor.
DR. R. H. L. BIBB, Associate Editor.

CONTRIBUTING EDITORS:

Dr. H. Sheridan Baketel, New York, editor *Medical Times;* Dr. G. Henri Bogart, Paris, Ill.; Dr. M. M. Carrick, Dallas, Texas; Dr. Edward H. Carey, Dean Medical Department, Baylor University, Dallas, Texas; Dr. W. L. Crosthwaite, Waco, Texas; Dr. T. D. Crothers, Hartford, Conn.; Dr. Oscar Dowling, New Orleans, President Louisiana State Board of Health; Havelock Ellis, M. D., London, Eng.; Dr. May Agnes Hopkins, Dallas, Texas; Dr. A. W. Fly, Galveston, Texas; Dr. G. B. Foscue, Waco, Texas; Dr. E. S. Goodhue, Holualoa, Kona, Hawaii; Dr. Joe Gilbert, Austin, Texas; Dr. Charles H. Hughes, St. Louis, editor *Alienist and Neurologist;* Dr. George H. Lee, Galveston, Texas; Dr. John T. Moore, Houston, Texas; Dr. Frank Paschal, San Antonio, Texas; Dr. John Preston, Austin, Texas, Superintendent State Lunatic Asylum; Dr. G. Wilse Robinson, Kansas City, Mo.; Dr. Bacon Saunders, Fort Worth, Texas; Dr. Allen J. Smith; Philadelphia, Pa., Medical Department, University of Pennsylvania; Dr. Z. T. Scott, Austin, Texas; Dr. Walter S. Sutton, Kansas City, Mo.; Dr. A. E. Sweatland, Nacogdoches, Texas; Dr. John S. Turner, Dallas, Texas; Dr. Charles Venable, San Antonio, Texas.

---

### It Isn't Your Town—It's You.

If you want to live in the kind of a town
Like the kind of a town you like,
You needn't slip your clothes in a grip
And start on a long, long hike.
You'll only find what you left behind,
For there's nothing that's really new.
It's a knock at yourself when you knock your town.
It isn't the town—it's you!

Real towns are not made by men afraid
Lest somebody else gets ahead.
When every one works and nobody shirks
You can raise a town from the dead.
And if while you make your personal stake
Your neighbors can make one, too,
Your town will be what you want to see,
It isn't the town—it's you!

—*Clipped.*

